# Enhanced Supercapacitor and Cycle-Life Performance: Self-Supported Nanohybrid Electrodes of Hydrothermally Grown MnO_2_ Nanorods on Carbon Nanotubes in Neutral Electrolyte

**DOI:** 10.3390/ma17164079

**Published:** 2024-08-16

**Authors:** Soraya Bouachma, Xiaoying Zheng, Alonso Moreno Zuria, Mohamed Kechouane, Noureddine Gabouze, Mohamed Mohamedi

**Affiliations:** 1 Centre Énergie, Matériaux et Télécommunications (EMT), Institut National de la Recherche Scientifique (INRS), 1650 Boulevard Lionel Boulet, Varennes, QC J3X 1S2, Canada; bouachmasouraya@crtse.dz (S.B.);; 2 Laboratory of Material Physics, Faculty of Physics, University of Science and Technology Houari Boumediene (U.S.T.H.B.), P.O. Box 32, El-Alia, Bab Ezzouar, Algiers DZ-16111, Algeria; 3 Centre de Recherche en Technologie des Semi-Conducteurs Pour l’Énergétique (CRTSE), Bd Frantz Fanon, P.O. Box 140, Alger-7 Merveilles, Algiers DZ-16038, Algeria

**Keywords:** manganese oxide nanorods, carbon nanotubes, hydrothermal, free-standing electrode, synergistic effect, supercapacitors

## Abstract

Efficient and sustainable energy storage remains a critical challenge in the advancement of energy technologies. This study presents the fabrication and electrochemical evaluation of a self-supporting electrode material composed of MnO_2_ nanorods grown directly on a carbon paper and carbon nanotube (CNT) substrate using a hydrothermal method. The resulting CNT/MnO_2_ electrodes exhibit a unique structural architecture with a high surface area and a three-dimensional hierarchical arrangement, contributing to a substantial electrochemical surface area. Electrochemical testing reveals remarkable performance characteristics, including a specific capacitance of up to 316.5 F/g, which is 11 times greater than that of conventional CP/MnO_2_ electrodes. Moreover, the CNT/MnO_2_ electrodes demonstrate outstanding retention capacity, exhibiting a remarkable 165% increase over 10,000 cycles. Symmetric supercapacitor devices utilizing CNT/MnO_2_ electrodes maintain a large voltage window of 3 V and a specific capacitance as high as 200 F/g. These results underscore the potential of free-standing CNT/MnO_2_ electrodes to advance the development of high-performance supercapacitors, which can be crucial for efficient and sustainable energy storage solutions in various industrial and manufacturing applications.

## 1. Introduction

Supercapacitors (SCs), sometimes referred to as electrochemical capacitors or ultracapacitors, are advanced energy storage devices that merge the high energy storage of traditional batteries with the rapid power delivery of conventional capacitors [1,2,3]. They are categorized based on their energy storage mechanisms as electrochemical double-layer capacitors (EDLCs) and pseudocapacitors. EDLCs utilize the double layer created by the separation of charges at the electrode–electrolyte interface, a process that is heavily influenced by the specific surface area and porosity of the electrode material. Alternatively, pseudocapacitors store electrical energy through rapid surface redox reactions. This Faradaic energy storage mechanism, relying solely on fast redox processes, enables significantly higher capacitance and energy density. In contrast, electrical double-layer capacitors (EDLCs), which use carbon-based materials, offer excellent cycling stability and high power density but have relatively lower capacitance and energy density due to their different operating mechanism. Pseudocapacitors display the opposite behavior, as the involvement of Faradaic redox reactions can lead to the accumulation of irreversible components during cycling, resulting in reduced performance over time. Hybrid supercapacitors, which combine the charge-storage mechanisms of both EDLCs and pseudocapacitors, achieve higher capacitance compared to EDLCs and an improved cycle life compared to pseudocapacitors. This combination enhances energy density without compromising power density.

Because of their low cost and high availability, carbon-based electrode materials, such as graphene, carbon nanosheets, nonporous carbon, carbon nanofibers, carbon nanotubes, activated carbon, and carbon aerogels, are widely used in EDLCs [4,5]. Carbon nanotubes (CNTs), owing to their high electrical conductivity, unique entangled network, and dominant porosity, are a very promising solution for overcoming the poor rate capability of metal oxide electrodes in supercapacitors [6]. In addition, CNTs can be grown on a variety of conductive substrates without the need for any binder or template [7]. This process decreases the interfacial resistance between the current collector and the active material, thereby simplifying the electrode fabrication and reducing its cost. Despite these advantages, EDLCs that utilize nanostructured carbon-based electrode materials, including CNTs, do not offer high energy density and high specific capacitance.

The limited energy density of EDLC-type supercapacitors can be significantly enhanced by using transition metal-based compounds as pseudocapacitive electrode materials. Various transition metal oxides (TMOs) such as RuO_2_, MnO_x_, NiO, Co_3_O_4_, V_2_O_5_, CuO, and ZnO, which can function as “proton condensers” in their hydrated forms, are being investigated as potential pseudocapacitor electrodes [8]. Indeed, the reversible reaction happening at the electrode–electrolyte interface helps to store more cations and thereby enhances the capacitance and the energy density. Owing to their being abundant in nature, being environmentally friendly, and having high theoretical specific capacitance, manganese oxides (MnO_x_s) are considered highly promising as electrode materials for next-generation pseudocapacitors [9]. However, similar to many transition metal oxides (TMOs), MnO_x_ suffers from low electrical conductivity, which restricts the pseudocapacitive redox reaction to a very thin surface layer. As a result, the full potential of MnO_x_ is realized only with an extremely thin layer of the oxide [10]. This restricts MnO_x_’s capacity, cyclic stability, and charge transfer rate characteristics. Combining porous and highly conductive CNTs with MnO_x_ would be an excellent strategy to maintain smooth electron and electrolyte ion pathways, providing SC devices with fast pseudocapacitance redox transitions, i.e., a much higher charge storage volume and an enhanced rate capability.

CNTs are typically synthesized using the chemical vapor deposition (CVD) method and can be grown with various catalysts. Conversely, MnO_x_ can be synthesized through a range of techniques, such as hydrothermal synthesis [11], precipitation [12], electrodeposition [13], plasma-enhanced CVD [14], or pulsed laser deposition (PLD) [15,16]. For the fabrication of CNT/MnO_x_ composites, methods include physical mixing [17], in situ hydrothermal processes [18], ball milling [19], electrophoresis [20], electrodeposition [21], redox reaction [22], co-precipitation [23], and microwave-assisted processes [24]. Despite extensive research on CNT/MnO_x_ composites, challenges remain in producing binder-free or self-supported electrodes for electrochemical energy storage applications [25]. Typically, composite electrodes are made with binders to ensure that the active material remains attached to the current collector during charge–discharge cycles. These binders are usually polymers with poor electrical conductivity, which can further reduce the overall conductivity of the electrode material. Conversely, directly growing the active material on the current collector through strong physical and/or chemical bonding enhances electronic conductivity and offers a larger exposed surface area for redox reactions. This not only maximizes the energy storage capability but also drastically reduces the cost of fabrication for large-scale industrial production.

This study reports on the electrochemical energy storage characteristics of binderless MnO_2_/CNT electrodes. The CNTs are grown onto a carbon paper (CP) substrate using chemical vapor deposition (CVD), whereas MnO_2_ nanorods are synthesized on the CNT substrate using the hydrothermal technique. The hydrothermal technique is chosen because it is inexpensive, ecological, and can be easily scaled up using several batch reactors in an industrial environment. The capacitive properties are assessed using both a three-electrode electrochemical cell and a custom-built symmetric supercapacitor (SC) device in a neutral 1 M Na_2_SO_4_ electrolyte solution. Neutral electrolytes are preferred for their benefits, including a relatively wide operating potential window, reduced corrosiveness, and enhanced safety, compared to the highly concentrated acid or alkaline electrolytes frequently used in supercapacitors [9].

## 2. Materials and Methods

### 2.1. Material Synthesis

*Synthesis of CNTs*-CNTs were synthesized on a CP substrate (Toray carbon paper TGP-H-60) at 700 °C by CVD with a ~5 nm nickel thin film as the catalyst, following a method described elsewhere [26]. In brief, a thin nickel layer was deposited on one side of the CP using the PLD technique. Subsequently, CNTs were grown on the CP/Ni sample via CVD with acetylene (as the carbon source), hydrogen, and argon (as the gas carriers) flowing at rates of 20, 100, and 140 sccm, respectively.

*Synthesis of MnO_x_ onto CP and CNTs/CP*—For the growth of MnO_x_, the hydrothermal method was employed using KMnO_4_ as the precursor. Typically, 1.67 mmol of KMnO_4_ (Sigma-Aldrich, St. Louis, MO, USA, 99%) was dissolved in 18.75 mL of ultrapure deionized water (Millipore Milli-Q, Merck, Burlington, MA, USA, resistivity 18.2 MΩ·cm) and stirred for 15 min until fully dissolved. Following this, 0.42 mL of concentrated hydrochloric acid (HCl, Sigma-Aldrich, 37%) was added to the solution, which was then stirred continuously for 2 min. Next, a 15 mm × 30 mm piece of CP or CNT sample was placed into a 25 mL Teflon-lined stainless-steel autoclave, and the KMnO_4_-HCl solution was carefully transferred into the autoclave. The autoclave was then sealed, placed in an oven, and heated to 140 °C for 12 h. After the synthesis was complete, the reactor was allowed to cool to room temperature. The CP/MnO_x_ and CNT/MnO_x_ samples were then removed, thoroughly rinsed with deionized water, and annealed in air at 300 °C for 1 h.

### 2.2. Materials Analysis

The surface morphology of the prepared samples was analyzed using a scanning electron microscope (SEM, TESCAN VEGA3, Brno, Czech Republic) operating at 20.0 kV. Details of the XRD, XPS, and Raman characterization of the samples can be found in our previous publication [26].

### 2.3. Electrochemical Measurements

The electrochemical performance was evaluated using an Eco Chemie PGSTAT302 potentiostat/galvanostat (Metrohm Autolab, Utrecht, The Netherlands). Measurements were performed at room temperature with a three-compartment electrochemical cell. This setup included a platinum coil as the counter electrode, an Ag/AgCl reference electrode (in 4 M KCl (aq)), and a rectangular CP/MnO_x_ or CNT/MnO_x_ electrode as the working electrode. To minimize the impact of the ohmic drop, the reference electrode was positioned close to the working electrode, separated by a Login capillary. The electrolyte employed was a 1 M Na_2_SO_4_ aqueous solution, which was degassed by passing argon through it for 20 to 30 min prior to each measurement to remove dissolved oxygen.

CV measurements were performed at scan rates ranging from 5 to 1000 mV/s. The specific capacitance, derived from the CV curves, was calculated using the formula *C_p_* = *Q*/(2*m* × *DV*), where *Q* represents the voltametric charge obtained by integrating the oxidation or the reduction areas of the CV curve, *m* (in grams) is the mass of the active material (CP, CNT, or MnO_2_) on the working electrode, and *DV* is the potential window of the CV. Cycling stability was studied through CV measurements at 200 mV/s over 10,000 continuous cycles.

## 3. Results and Discussion

### 3.1. Materials Characterization

**Figure 1:**
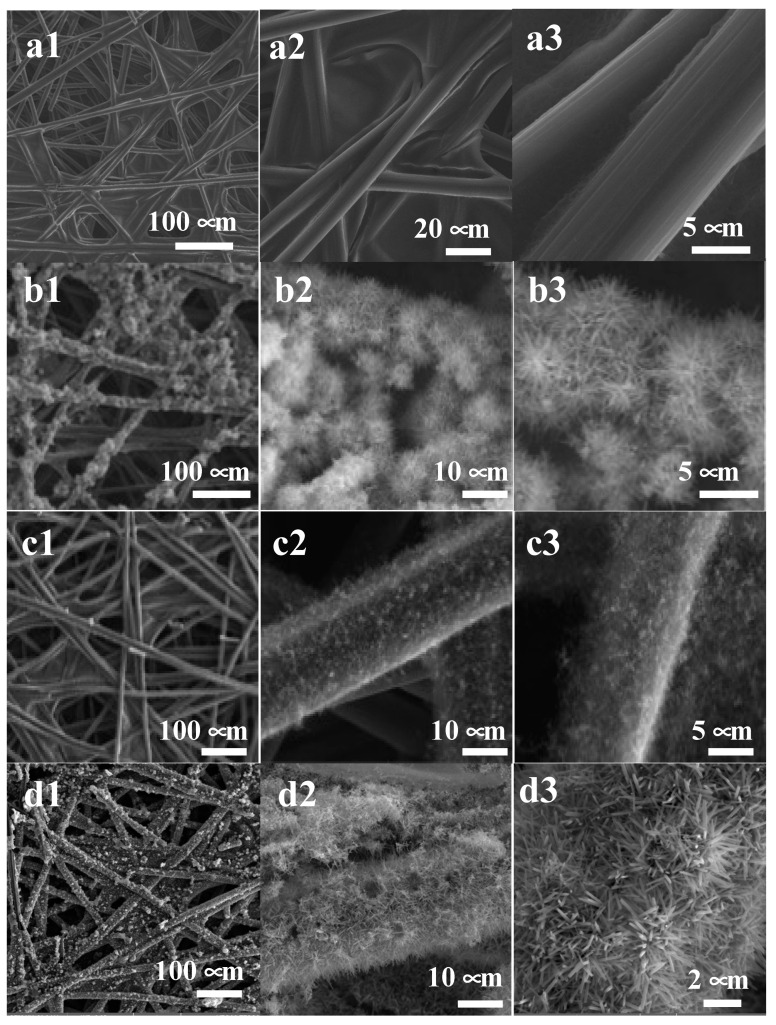
SEM images at varying magnifications are provided for the following samples: (**a1**–**a3**) CP; (**b1**–**b3**) CP/MnO_x_; (**c1**–**c3**) CP/CNT; and (**d1**–**d3**) CNT/MnO_x_.

The surface morphologies of CP, CP/MnO_x_, CP/CNT, and CP/CNT/MnO_x_, as examined by the SEM, are shown at various magnifications in Figure 1a–d. It is evident that both the microfibers of the CP substrate (Figure 1(b1–b3)) and the CNTs (Figure 1(d1–d3)) are uniformly covered with a high density of hierarchical MnO_x_ nanorod arrays, which have gaps at the top. This 3-D open porous structure is anticipated to facilitate electrolyte penetration, which improves ion transport and provides a smooth pathway for electron flow, leading to a high charge–discharge rate. The estimated average diameters of the MnO_x_ nanorods were approximately 200 nm for the CP/MnO_x_ and around 155 nm for the CNTs/MnO_x_. The detailed XRD, XPS, and Raman characterization of the samples can be found in our previous publication [26]. In summary, XRD analysis indicated that the MnO_x_ grown on both CP and CNT substrates was cryptomelane KMn_8_O_16_, a type of tetragonal α-MnO_2_, in which K^+^ ions are incorporated into some of the 2 × 2 MnO_6_ tunnel structures. Raman analysis further confirmed the tetragonal α-MnO_2_ characteristics (cryptomelane type). Additionally, XPS results revealed that the Mn 2p core level exhibited two peaks separated by a spin energy difference of 11.8 eV, consistent with the MnO_2_ structure.

### 3.2. Electrochemical Performance

**Figure 2:**
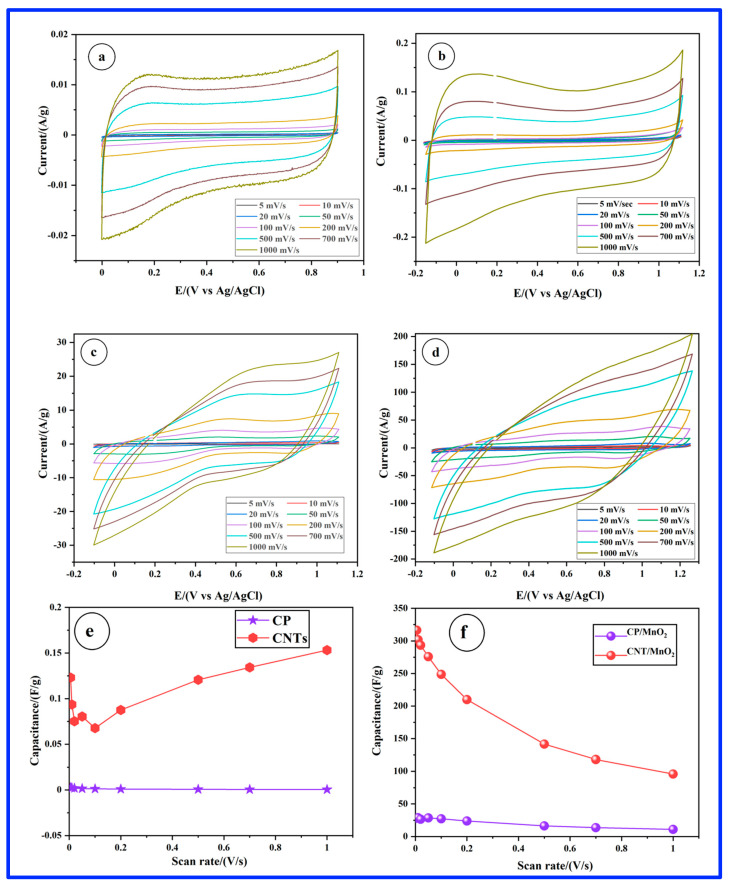
Cyclic voltammetry with increasing scan rates in a three-electrode system employing a 1 M Na_2_SO_4_ electrolyte solution for the following samples: (**a**) CP, (**b**) CNT, (**c**) CP/MnO_2_, and (**d**) CNT/MnO_2_. (**e**,**f**) Specific capacitances as a function of the scan rates.

The electrochemical properties of as-prepared samples were initially characterized by CV in a three-electrode cell employing a 1 M Na_2_SO_4_ solution as the electrolyte. Figure 2a,b present CVs recorded for the CP and CP/CNT electrodes at scan rates varying from 5 to 1000 mV/s. The CV curves for the CP and CNT electrodes display a nearly symmetrical rectangular shape, indicative of electrical double-layer capacitance, in which all charges are stored on the surface of the materials. For the CP electrode, the capacitive behavior is observed over a 0.9 V potential window, whereas with the presence of CNTs, the potential window extends over a larger range of 1.25 V. At both CP and CP/CNT electrodes, the current increases with an increasing scan rate. The specific capacitances derived from the CVs of Figure 2a,b, plotted as a function of scan rate, are presented in Figure 2e. For the CP electrode, the specific capacitance decreases as the scan rate increases. In contrast, the CP/CNT electrode shows a slight recovery in specific capacitance at higher scan rates. Despite this, the specific capacitance of the bare CP/CNTs remains relatively low, peaking at approximately 0.16 F/g at 1 V/s. These low values are anticipated for CNTs due to their hydrophobic nature and the absence of any surface treatment.

In comparison, the CP/MnO_2_ (Figure 2c) and CP/CNT/MnO_2_ (Figure 2d) electrodes also display fairly rectangular CV curves, suggesting rapid and reversible Faradaic reactions and optimal capacitive performance [27,28]. However, slight distortions appear as the scan rate increases, reflecting polarization due to heightened transport resistance. This pseudocapacitance primarily arises from the surface adsorption of Na^+^ cations (surface redox reactions) and their integration into the MnO_2_ matrix. It can be further noticed that the capacitive potential window has been expanded either with CP/MnO_2_ (1.2 V) or CNT/MnO_2_ (1.35 V) as compared to the 0.9 V with CP and 1.25 V with CP/CNT, respectively. Figure 2f illustrates the rate capability of carbon-based MnO_2_ electrodes by showing how specific capacitance varies with the scan rate. It is evident that specific capacitance decreases for all electrodes as the scan rate increases. A notable observation is the high specific capacitance of the CNT/MnO_2_ electrode, which reaches 316.5 F/g at 5 mV/s, a value 11 times greater than the value delivered by the CP/MnO_2_ electrode (28 F/g) at the same scan rate. This significant capacitance for CNT/MnO_2_ is attributed to the unique nanorod structure with highly exposed active surfaces that facilitate rapid electrolyte diffusion and Na^+^ ion transfer, as well as the enhanced conductivity provided by the CNTs, which improves electron collection. Meanwhile, the specific capacitance decreased significantly at scan rates higher than 200 mV/s. This behavior is characteristic of TMOs and results from the reduced diffusion of electrolyte ions into the active material matrix at higher scan rates [29]. It is also crucial to highlight that the specific capacitance observed in our study is attributable entirely to the MnO_2_ component, as the contribution from the CNTs is minimal (0.16 F/g). This underscores the fact that the hydrothermal technique effectively maximizes the utilization of MnO_2_ in the composite.

**Figure 3:**
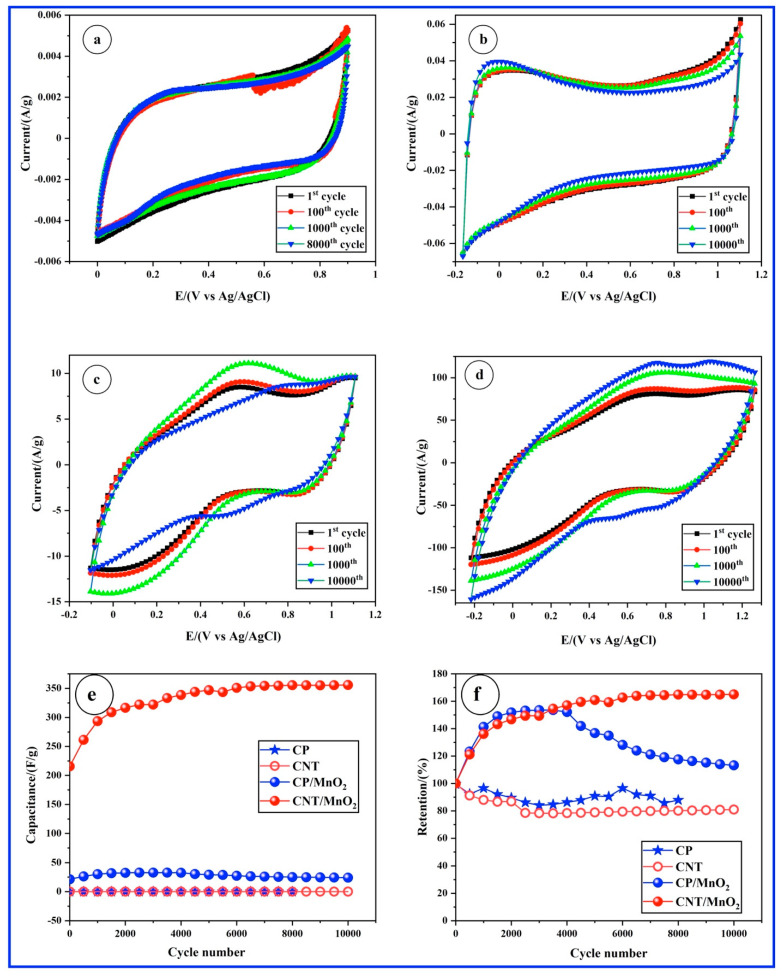
Cycle-life performances assessed with CV at a scan rate of 200 mV/s in a 1 M Na_2_SO_4_ electrolyte solution for the following samples: (**a**) CP, (**b**) CNT, (**c**) CP/MnO_2_, and (**d**) CNT/MnO_2_. (**e**) Specific capacitances as a function of cycle number. (**f**) Specific capacitance retention, C/C_0_.

The cycle-life performances of the four materials were tested over 10,000 cycles using CV at a scan rate of 200 mV/s. Figure 3 shows the CVs for selected cycles, including the 1st, 100th, 1000th, 8000th, and/or 10,000th cycles. In Figure 3a,b, the CV curves of the CP and CP/CNT electrodes exhibit remarkable stability throughout the numerous charge–discharge cycles. However, for the CP/MnO_2_ and CNT/MnO_2_ electrodes, oxidation and reduction processes occur during CV cycling, attributed to redox reactions of surface Mn ions (Figure 3c,d). Figure 3e presents the specific capacitance derived from Figure 3a–d plotted against the number of CV cycles. The capacitance of CP and CNT electrodes remains relatively stable over 10,000 cycles, whereas that of CP/MnO_2_ and CNT/MnO_2_ electrodes increases during cycling. Figure 3e shows the retention of specific capacitance (C/C_0_) over 10,000 continuous charge–discharge cycles, where C_0_ denotes the specific capacitance from the initial cycle. The CP and CNT electrodes show a loss of about 12% and 19% of their initial specific capacitance, respectively, while the CP/MnO_2_ and CNT/MnO_2_ electrodes demonstrate an outstanding retention capacity, with a remarkable 165% increase over 10,000 cycles, which is particularly notable for the latter electrode. This increase in specific capacitance of MnO_2_ CV cycling stems from various factors. MnO_2_ demonstrates pseudocapacitive behavior (as shown in Figure 2c,d), wherein charge storage occurs via Faradaic redox reactions at the electrode–electrolyte interface [30]. Throughout cycling, active sites on the MnO_2_ surface may become more accessible or undergo activation, thereby amplifying redox reactions and augmenting capacitance [31]. Structural alterations induced by cycling, like the formation of more porous structures or the exposure of additional active sites in MnO_2_, can also contribute to increased capacitance by providing a larger surface area for charge storage [32]. Additionally, cycling may enhance the accessibility of ions within the electrolyte to the MnO_2_ electrode, promoting more efficient ion transport and charge storage, thus boosting capacitance. Moreover, the incorporation of carbon nanotubes not only enhances the conductivity of MnO_2_ (and thus its capacitance) but also imparts mechanical stability to it [33].

**Figure 4:**
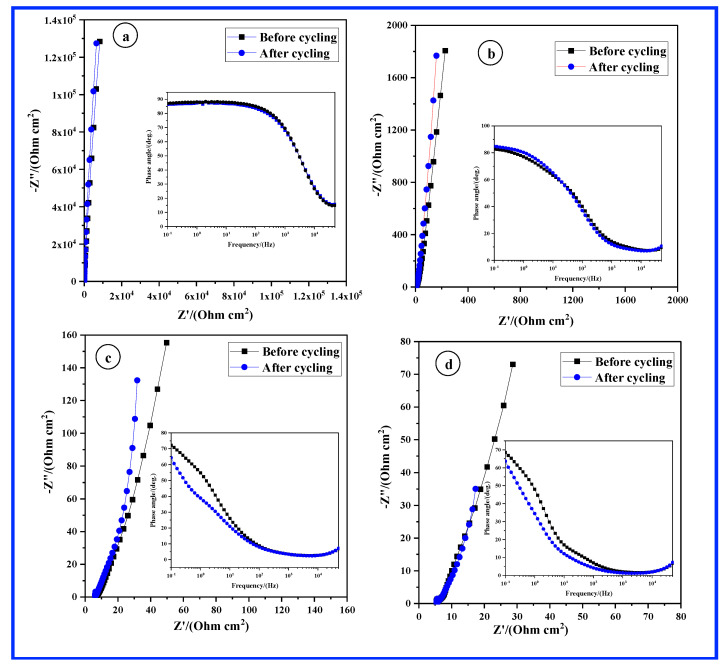
Nyquist and Bode plots depicting EIS data obtained before and after CV cycle-life testing for the following samples: (**a**) CP, (**b**) CNT, (**c**) CP/MnO_2_, and (**d**) CNT/MnO_2_. Insets display Bode plot representations showcasing phase angle vs. frequency.

In Figure 4, EIS spectra were recorded both before and after the CV cycling depicted in Figure 3a–d. The absence of a noticeable semi-circle in the Nyquist plots indicates an exceptionally fast charge transfer, signifying that the electrode material enables highly efficient charge transfer between the electrode and the electrolyte. This characteristic is particularly advantageous for supercapacitors, as it demonstrates the electrode’s capability to handle rapid charge–discharge cycles with minimal resistance. Additionally, the EIS spectra for both CP and CNT display a linear trend with a phase angle nearing 90 degrees, which is indicative of typical electrochemical double-layer capacitor behavior. Conversely, the EIS spectra of CP/MnO_2_ and CNT/MnO_2_ deviate from this linear behavior, showing a phase angle of less than 80 degrees. This deviation suggests the presence of pseudocapacitive behavior, reflecting the contribution of Faradaic reactions to the overall capacitance of these materials. Furthermore, the resistance at very low frequencies is ordered as CNT/MnO_2_ (28.7 W cm^2^) < CP/MnO_2_ (49.6 W cm^2^) < CNT (227 W cm^2^) < CP (8.2 kW cm^2^). This order of resistance at very low frequencies reflects the varying degrees of conductivity and pseudocapacitive properties of the different electrode configurations. The CNT/MnO_2_ combination exhibits the lowest resistance at very low frequencies because of CNTs’ excellent electrical conductivity and MnO_2_’s high specific capacitance. Therefore, the CNT/MnO_2_ composite would offer the most favorable conditions for charge transfer at very low frequencies, resulting in the lowest resistance. In addition, after the CV cycling, there is minimal change in the EIS spectra across all samples, as shown in Figure 4. This indicates that the electrode configurations maintain their structural and electrical integrity even after undergoing repeated cycles of charge and discharge during CV cycling.

### 3.3. Symmetric Supercapacitor Performance

**Figure 5:**
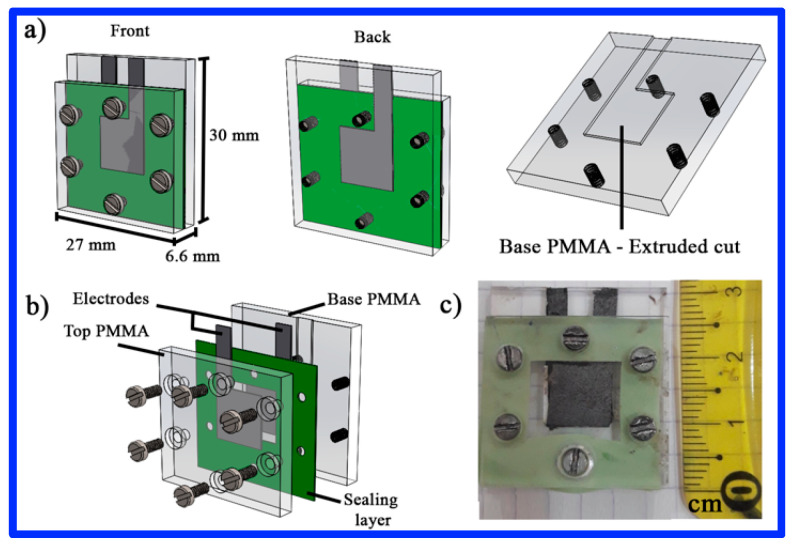
Homemade SC device. (**a**) Front view and (**b**) back view. (**c**) Photograph of the fabricated SC device.

The performance of CP/MnO_2_ and CNT/MnO_2_ nanorod electrodes was further investigated in a custom-made supercapacitor (SC) device, as illustrated in Figure 5. Through computational design, a configuration for the SC device was developed to determine the dimensions of each component and streamline the assembly process and material fabrication. Constructed from polymethyl methacrylate (PMMA), the SC device comprised a top plate and a base plate. The base plate featured an extruded cut at its center to accommodate an electrode. After finalizing the design, the complete SC device was assembled. Micromachining of both PMMA plates was performed using a Nomad 883 Carbide 3-D Computer Numerical Control (CNC) machine (Torrance, CA, USA). The SC device was sealed with a mixture of two epoxies (Silastic), specifically RTV Silicone Rubber Base^®^ and RTV Silicone Rubber Curing Agent^®^, combined in a 9:1 ratio. This design was implemented in the Silhouette Studio^®^ program for vectorization and manufacturing with a Grapthec America Inc 2D cutting plotter. A conductive carbon paper served as the current collector adjacent to each electrode (1 cm^2^). A filter paper (Whatman™) soaked in a 1 M Na_2_SO_4_ solution was placed between the positive and negative electrodes, and the SC device was assembled using six screws.

**Figure 6:**
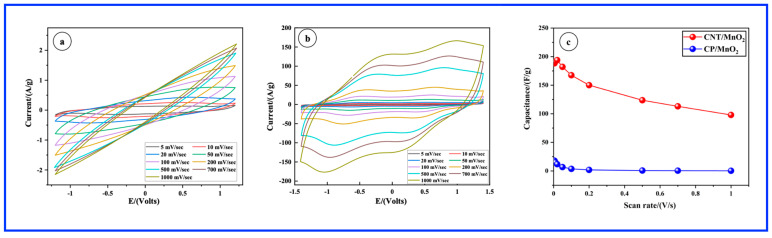
Cyclic voltammetry with increasing scan rates in a symmetric supercapacitor device containing a 1 M Na_2_SO_4_ electrolyte. (**a**) CP/MnO_2_ and (**b**) CNT/MnO_2_. (**c**) Specific capacitances extracted from Figure 6a,b.

Subsequent electrochemical evaluations were carried out using the SC device. Figure 6a,b illustrate CVs obtained at increasing scan rates for CP/MnO_2_ and CNT/MnO_2_ electrodes, respectively. It is evident from the CV profiles of CP/MnO_2_ that distortion occurs at high scan rates, approaching resistive behavior (Figure 6a). Such distortion may arise from resistance effects, wherein high scan rates induce increased ohmic resistance within the SC device, particularly at the electrode–electrolyte interface. This resistance can impact the kinetics of electrochemical reactions and contribute to distortions in the voltammograms. In contrast, the SC device featuring CNT/MnO_2_ electrodes demonstrates improved performance and reduced distortion in CVs, even at high scan rates (Figure 6b). This improvement can be attributed to several factors specific to the CNT/MnO_2_ composite structure. CNTs exhibit excellent electrical conductivity, facilitating rapid electron transport within the electrode material. Consequently, this can alleviate the effects of high scan rates on the kinetics of electrochemical reactions, thus reducing distortion in the voltammograms. Additionally, the porous nature of the CNT/MnO_2_ composite enhances ion diffusion throughout the electrode material, ensuring better electrolyte accessibility to the active sites on the MnO_2_ surface. This, in turn, mitigates diffusion limitations that could lead to distortions in the voltammograms. Furthermore, the SC device demonstrates a large capacitive potential window of 3 V, regardless of whether CP/MnO_2_ or CNT/MnO_2_ electrodes are employed. Figure 6c illustrates the rate capability by showing how specific capacitance changes with scan rates. It is noted that the specific capacitance of all electrodes declines as the scan rate increases. Notably, Figure 6c highlights the remarkable specific capacitance of CNT/MnO_2_, reaching close to 200 F/g at 5 and 10 mV/s. This value is approximately eight times higher than that achieved by CP/MnO_2_ (25 F/g) at the same scan rates.

In considering the broader theoretical framework, it is acknowledged that incorporating perspectives on low-carbon energy technologies, such as those discussed by Grigorios L. Kyriakopoulos in relation to sustainable energy systems [34], could provide valuable context for the findings presented. While the focus of this study remains the synthesis and electrochemical performance of MnO_2_/CP and MnO_2_/CNT electrodes for supercapacitors, future research may benefit from integrating such broader discussions to enrich the understanding of how these findings relate to wider energy system challenges and opportunities.

## 4. Conclusions

The hydrothermal growth method employed in this study yields a remarkable architecture, with MnO_2_ nanorods densely arrayed on a carbon paper and CNT substrate. The resulting structure boasts exceptional surface characteristics, including a high surface area and a three-dimensional hierarchical arrangement. Such features significantly augment the electrochemical surface area, paving the way for enhanced performance in supercapacitor applications. Notably, the self-supporting structure of CNT/MnO_2_ electrodes showcases outstanding electrochemical performance, with a specific capacitance as high as 316.5 F/g. This represents a substantial 11-fold increase compared to the CP/MnO_2_ counterpart, which registers at 28 F/g. Equally impressive is the longevity of the CNT/MnO_2_ electrodes, which exhibit a remarkable 165% increase in retention capacity over 10,000 cycles.

Further evaluations employing symmetric supercapacitor devices underscore the robustness of CNT/MnO_2_ electrodes, which sustain a large voltage window of 3 V and retain a specific capacitance as high as 200 F/g. This strong performance can be attributed to the synergistic effects of the unique nanorod forest structure, which facilitates rapid Na^+^ ion transfer, and the enhanced conductivity facilitated by the incorporation of CNTs. Notably, the integration of CNTs not only boosts the conductivity of MnO_2_, thereby augmenting its capacitance, but also fortifies its mechanical stability, ensuring long-term reliability in energy storage applications. The exceptional electrochemical performance and durability exhibited by the free-standing CNT/MnO_2_ electrodes make them highly promising for supercapacitor applications.

## Data Availability

The original contributions presented in the study are included in the article, further inquiries can be directed to the corresponding author.
